# Perioperative Nursing Process: reflection on updating the SAEP nomenclature

**DOI:** 10.1590/1980-220X-REEUSP-2025-0165en

**Published:** 2025-10-20

**Authors:** Juliana Rizzo Gnatta, Vanessa de Brito Poveda, Elaine Ribeiro, Erika Christiane Marocco Duran, Camila Takao Lopes, Cassiane de Santana Lemos, Marla Andréia Garcia de Avila, Suzimar de Fátima Benato Fusco, Taysa de Fátima Garcia, Rita Catalina Aquino Caregnato

**Affiliations:** 1 Universidade de São Paulo, Escola de Enfermagem, Departamento de Enfermagem Médico-Cirúrgica, São Paulo, SP, Brazil.; 2 Universidade Estadual de Campinas, Faculdade de Enfermagem, Campinas, SP, Brazil.; 3 Universidade Federal de São Paulo, Escola Paulista de Enfermagem, São Paulo, SP, Brazil.; 4 Universidade Estadual Paulista, Faculdade de Medicina de Botucatu, Departamento de Enfermagem, Botucatu, SP, Brazil.; 5 Universidade Federal de Minas Gerais, Belo Horizonte, MG, Brazil.; 6 Universidade Federal de Ciências da Saúde de Porto Alegre, Departamento de Enfermagem, Porto Alegre, RS, Brazil.

**Keywords:** Nursing, Perioperative Nursing, Perioperative Period, Nursing Process, Nursing Care

## Abstract

**Objective:**

To reflect on the relevance of the Systematization of Nursing Care in the perioperative practice (*SAEP*) of nurses, comparing it to Resolution No. 736 of 2024 of the Brazilian Federal Nursing Council (COFEN) and proposing the update of its nomenclature to ensure compliance with the aforementioned Resolution.

**Method:**

This is a reflective study in which experts in perioperative care and/or in Nursing Process (NP) met to discuss the framework named SAEP, adopted by the Brazilian perioperative nurses since the 1990s. Historical aspects of the conceptual framework “Perioperative Nursing Systematics” are discussed, addressing its relevance in the perioperative nurses’ practice and comparing it with Resolution No. 736/2024 of the Federal Nursing Council.

**Results:**

The SAEP framework proposed in the 1990s is still current in perioperative nursing practice, as it covers the stages of the NP in its entirety. Considering its importance, current status, and applicability in the perioperative nursing practice, parallels are established with the stages of the NP described in Resolution No. 736, suggesting a new nomenclature: Perioperative Nursing Process (*PEP*).

**Conclusion:**

An update of the nomenclature from SAEP to PEP is recommended, by aligning the SAEP conceptual framework, COFEN Resolution No. 736 of 2024, and the “Nursing Process” terminology adopted globally.

## INTRODUCTION

In January 2024, the Brazilian Federal Council of Nursing (COFEN) published Resolution 736, which addresses the implementation of the Nursing Process (NP) in every socio-environmental context where nursing care occurs^(1)^. This publication resulted in the revocation of COFEN Resolution 358/2009, which regulated the Systematization of Nursing Care (*SAE*) and the implementation of the NP in public or private settings where professional Nursing care is provided, and provided other provisions^(2)^.

The revoked Resolution, 358/2009, defined *SAE* as the organization of professional work in terms of method, personnel and instruments, enabling the operationalization of the NP. However, all of its content referred to the NP, defining it as “a methodological instrument that guides professional nursing care and the documentation of professional practice”^(2)^.

Because *SAE* is a managerial/organizational attribute, i.e., dependent on nursing management, in addition to being considered an immature concept^(3)^, the working group appointed by COFEN to draft Resolution 736/2024 decided not to address it. The current Resolution presents the NP as a methodology to guide nurses’ work, based on critical, reflective thinking and clinical judgment, enabling the implementation of technical-scientific knowledge in care practice directed toward the care of the individual, the community and groups, guiding care management^(1)^.

Prior to the publication of COFEN Resolution 358/2009^(2)^, Jouclas and Castellanos devised the framework called “Perioperative Nursing Care Systematics” (SAEP) in 1990, with the proposal of a holistic assessment of the patient, providing individualized care focused on the actual needs of the surgical patient^(4)^. This framework was later nationally referred to as “Systematization of Perioperative Nursing Care”^(5)^.

The Brazilian Perioperative Nursing field is familiar with the term SAEP, implementing this framework to provide comprehensive care to surgical patients across all phases of the perioperative period (preoperative, intraoperative, and postoperative) in a continuous, participatory, individualized, documented and evaluated manner^(4)^. However, given the conceptual confusion between SAE and NP over the decades, in light of the new Resolution No. 736/2024, it is relevant to consider updating the nomenclature “SAEP”. The conceptual confusion between SAE and NP has led professionals to use the terms interchangeably, impairing their understanding and differentiation^(6,7,8)^. In clinical practice, this confusion projects NP not as an intellectual guide or the application of the scientific method in nursing practice, but as a bureaucratic set of tasks or documentation for which adequate resources are often lacking. This perception may discourage clinical nurses from appropriating the adequate use of the NP method and discourage managers from seeking sufficient resources for its implementation.

It should be noted that the expression *SAE* exists only in Brazil, whereas internationally, the term “*Nursing Process”* is used, an expression also indexed as a descriptor in scientific databases. “The concept of Nursing Care Systematization (SAE) emerged in Brazil, with the premise that it would be the context in which the operationalization of the NP would take place”^(9)^. In this regard, the current COFEN Resolution, by removing the term SAE from its text and focusing solely on the NP, prompted the following question among nurses with perioperative expertise: what would be the recommended nomenclature to represent the elements encompassed by SAEP, as used in the Brazilian perioperative nursing field? In response, specialists convened to discuss this matter and defined as their objective to reflect on the relevance of *SAEP* in the perioperative practice of nurses, comparing it to Resolution No. 736 of 2024 of COFEN and proposing the update of its nomenclature to ensure compliance with the aforementioned Resolution.

## METHOD

### Study Design

This is a reflective study developed by a group of nurses with expertise in perioperative care and/or the Nursing Process. The group includes members of the Board of Directors of the Brazilian Association of Operating Room Nurses, Anesthetic Recovery and Material and Sterilization Center (SOBECC) for the 2023–2024 term, as well as are members of the Nursing Process Research Network (RePPE) and the Nursing Practice Systematization Commission (COMSISTE) of the Brazilian Nursing Association (ABEn). The group discussed historical aspects of the conceptual framework SAEP, proposed by Castellanos and Jouclas^(4)^ and adopted by Brazilian perioperative nurses, addressing its relevance in the nurse’s perioperative practice and comparing it with Resolution No. 736 of COFEN, published in 2024^(1)^. Finally, the group proposes the adoption of a nomenclature to be aligned with the current Resolution, replacing SAEP.

### Ethical Aspects

As this is a reflective study that does not involve human beings, this manuscript does not require approval from a Human Research Ethics Committee or and Informed Consent Form.

## RESULTS

Brazilian nursing professionals, when using the SAEP terminology, considered that this nomenclature expressed the NP throughout the entire perioperative period, highlighting the intrinsic interconnection between the three phases: preoperative, intraoperative, and postoperative. Within the SAEP framework, the NP would be used as a tool to equip nurses to provide continuous care by identifying individual needs, recognizing human responses, and defining specific care at each stage of the perioperative period.

The SAEP framework recommends that the “Preoperative Assessment” be conducted through a preoperative visit carried out by the operating room (OR) nurse at some point during the 24 hours preceding the anesthetic-surgical procedure^(4)^. Therefore, from the moment the preoperative visit begins, the Perioperative Nursing Process actually begins. This stage corresponds to the first stage of the NP proposed by Resolution 736/2024, “nursing assessment”^(1)^, and involves performing history taking, physical examination and direct observation, reviewing laboratory and imaging test results, and identifying the needs and specificities of care during the transoperative period.

Thus, the “preoperative assessment” stage begins with the OR nurse visiting the inpatient unit where the patient is in person, or connecting with the patient through an information and communication technology, such as telenursing. The perioperative nurse performs the assessment through history taking, physical examination and reviewing of test results, and documents all findings. This is followed by the second stage, referred in Resolution 736/2024 as “Nursing Diagnosis”. Based on the identification of problems, vulnerabilities or readiness to improve the patient’s health behaviors, the nurse identifies the nursing diagnoses that support the development of an individualized “Care Planning” to be implemented in the pre- and intraoperative periods, tailored to the specificities of the patient who will undergo the anesthetic-surgical procedure. This moment corresponds to the third stage of the NP, named “Nursing Planning”.

When admitting a patient who has already gone through a preoperative assessment in person or via telenursing, the nurse who performs the admission to the operating room receives information and analyzes all previously completed and documented NP steps. Furthermore, when admitting the patient, the nurse assesses and documents the patient’s status on the day of surgery, determines and documents whether to maintain and/or modify the identified nursing diagnoses, and initiates the “Care Implementation”, which corresponds to the fourth stage of the NP – “Nursing Implementation”.

At the end of the intraoperative period, the OR nurse documents the “Nursing Evaluation”, the fifth stage of the NP, and the patient is transferred to the Post-Anesthesia Care Unit (PACU). In the PACU, the patient is received by the unit nurse, who receives information from the interprofessional team and reviews all records of the NP stages completed, from the patient’s preoperative assessment through the conclusion of the intraoperative period. The nurse then applies all NP stages: re-assesses and documents the patient’s clinical condition, any new problems and vulnerabilities identified, defines or redefines the immediate post-operative period care plan through nursing prescription. Upon discharge from the PACU, the nurse performs the nursing evaluation and transfer is transferred to the destination unit, where the nursing professional from the respective unit will continue the care guided by the NP. It should be noted that patients undergoing surgeries that require critical care in the immediate postoperative period are transferred directly from the OR to the Intensive Care Unit, ensuring continuity of the same NP stages.

It should be noted that in the SAEP framework by Castellanos and Jouclas^(4)^, it is recommended that the OR nurse carry out the postoperative visit to reassess all proposed care and analyze the outcomes achieved throughout the perioperative period. During this visit, the patient’s level of satisfaction and any complications identified should be documented, such as evidence of injury due to inadequate surgical positioning.

**Figure 1:**
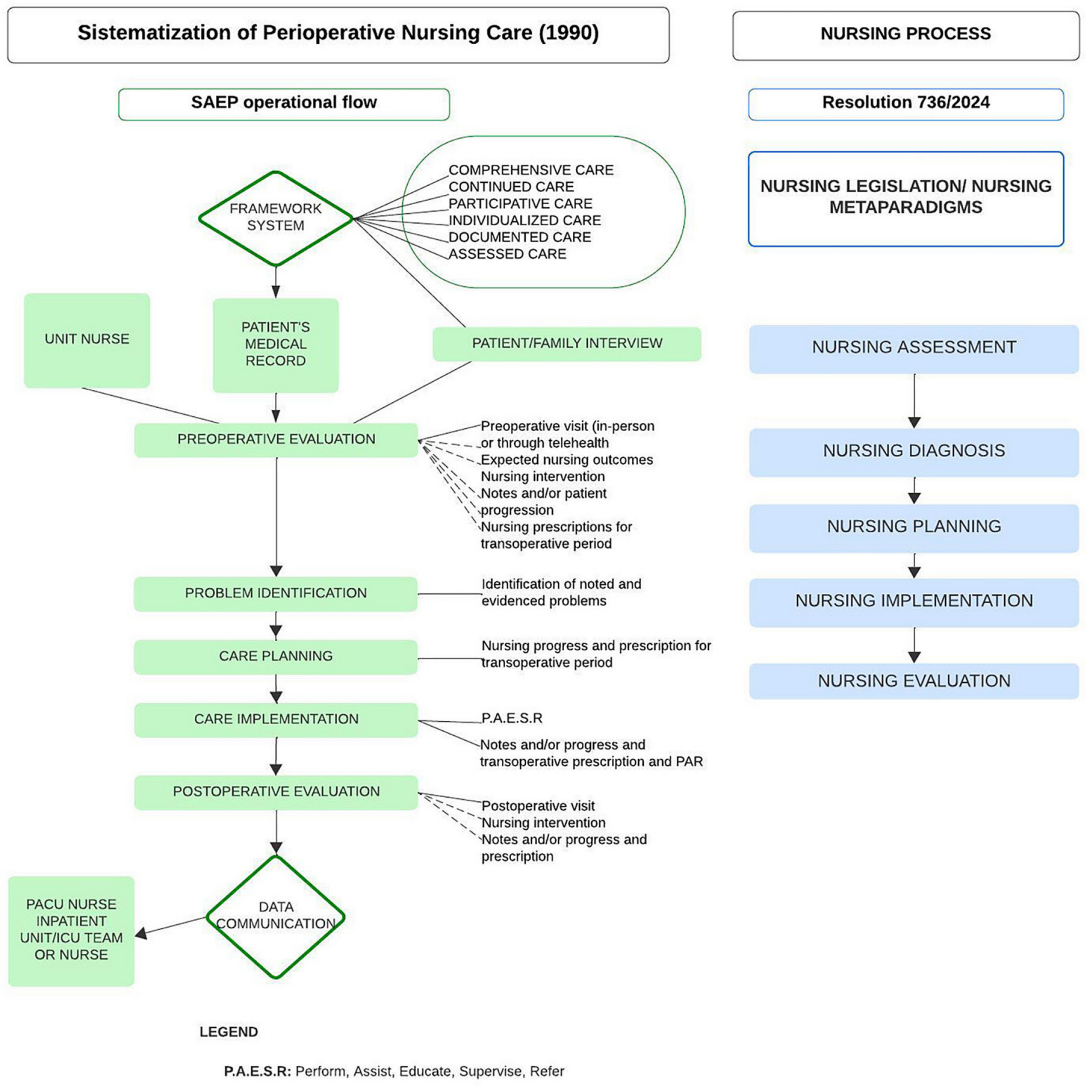
Mapping of the progress of the Perioperative Nursing Process in the SAEP framework^(4)^ and its correspondence with the Nursing Process in the COFEN Resolution 736/2024^(1)^. São Paulo, 2025.

From this perspective, it is important to clarify that the SAEP model proposed in the 1990s remains in use in perioperative nursing practice, as it encompasses all NP stages in their entirety, and emphasizes the need for a supporting theoretical framework, in alignment with the profession’s metaparadigms: environment, health, nursing, and person. Additionally, the managerial aspects of perioperative care are incorporated in this framework, as shown in Figure 1.

Figure 1 highlights the magnitude and complexity of the SAEP framework and its correspondence with the NP stages in the COFEN Resolution 736/2024.

The complete execution of the NP tailored to the patient’s needs enables a comprehensive and individualized assessment of those needs. This facilitates the organization of human and material resources in alignment with the specificities of the surgery, the demands of the patient and the needs of the surgical team. It also supports the nurse throughout the perioperative planning, beginning at the moment of care transfer. Notably, the systematic documentation of the NP stages enables monitoring care, clinical and financial indicators. From this perspective, it also highlights the relationship between the NP and the surgical patient safety.

**Figure 2:**
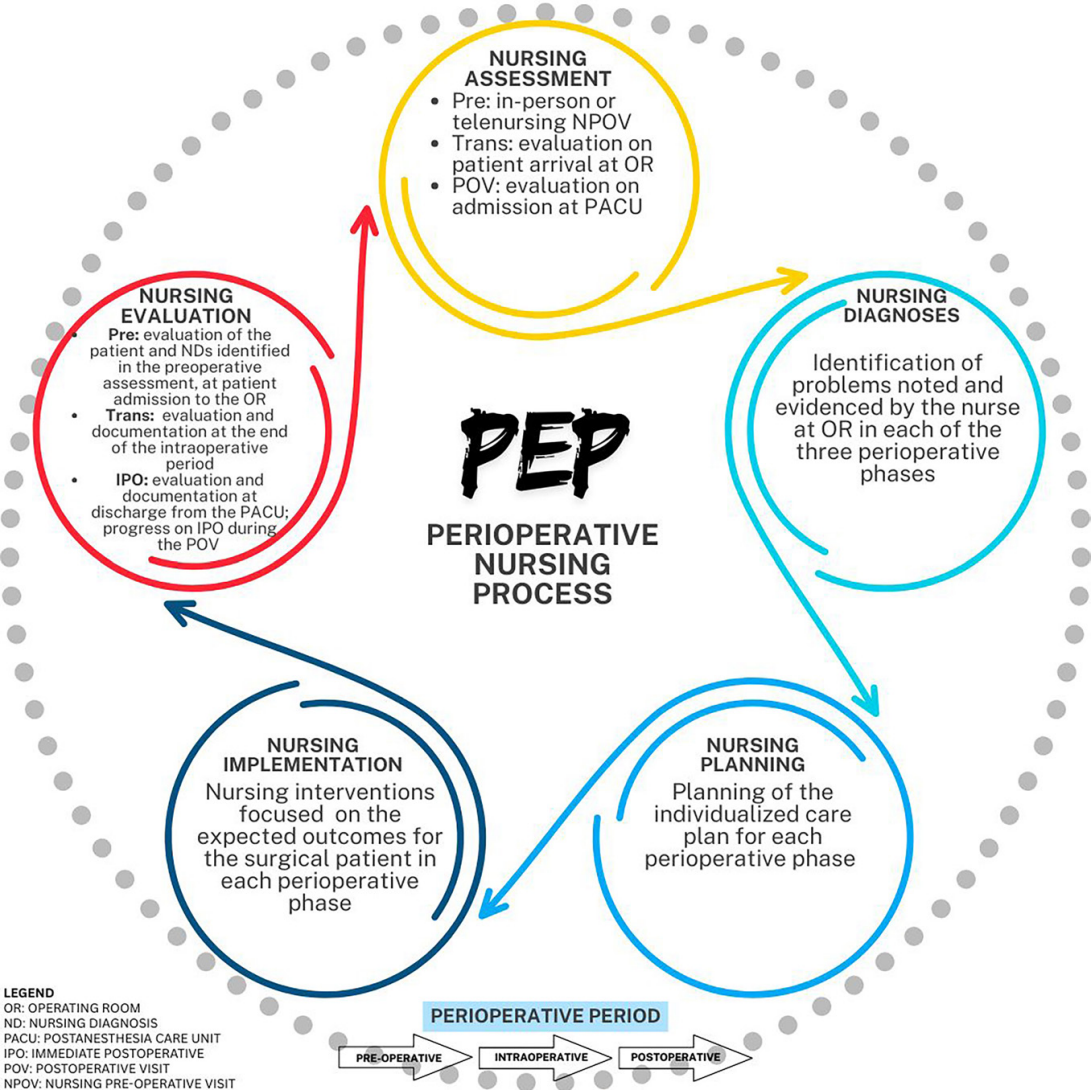
Perioperative Nursing Process (*PEP*) structure. São Paulo, 2025.

Considering this reflection, the adoption the term “Perioperative Nursing Process” (PEP) is proposed to replace SAEP, since PEP maintains consistency with the SAEP conceptual framework and is organized into five interrelated stages (Figure 2), which correspond to the NP stages, as shown in Figure 1.

As shown in Figure 2, the PEP must be implemented across all three phases of the perioperative period (pre-, trans- and post-operative), in a continuous and evaluative manner, ensuring comprehensive, participatory, continuous and individualized care for the surgical patient.

## DISCUSSION

Resolution No. 736^(1)^ determines that the NP must be implemented in every context in which nursing care is provided; therefore, it encompasses the care of surgical patients. The Resolution reinforces the five interrelated, interdependent, recurring and cyclical stages of the NP, namely: 1. “Nursing Assessment”, characterized by the collection of subjective (interview) and objective (physical examination, laboratory and imaging tests) data to obtain pertinent information about nursing care needs; 2. “Nursing Diagnosis”, which establishes the actual problems and conditions of vulnerabilities or readiness to improve health behaviors through clinical judgment of the information obtained in the previous stage; 3. “Nursing Planning”, when a care plan is developed aimed at the individual, family, community or special groups, shared with the care recipients and the nursing and healthcare teams; 4. “Nursing Implementation”, which includes the execution, by the nursing team, of interventions, actions and activities outlined in the care plan; 5. “Nursing Evaluation” which verifies the nursing outcomes achieved and identifies the health status of the individual, family, community and special groups, allowing the nurse to analyze and review the entire NP, returning to the first stage^(1)^. In clinical practice, the SAEP framework applies these five stages in a continuous and evaluative manner across the three perioperative stages (Figure 2).

The SAEP framework, proposed by Castellanos and Jouclas^(4)^, aligns with Resolution 736/2024^(1)^, since the “preoperative assessment” incorporates the data collection carried out during “nursing assessment” and, in preoperative period, includes the preoperative nursing visit; the “problem identification” corresponds to the definition of the “nursing diagnoses”; “care planning” is developed through the “nursing planning”; the “implementation of nursing care for the transoperative period” refers to the “nursing implementation” itself; and, finally, the “postoperative care evaluation” is equivalent to the “nursing evaluation”, a phase intended to evaluate planned and implemented care, including the “postoperative nursing visit” in the immediate postoperative period (Figure 1).

The PEP can be defined as the structuring axis of Perioperative Nursing care, a method that guides nurses’ critical thinking and clinical judgment in pre- and post-operative hospitalization units and ORs, directing the nursing team in the care of individuals, families, and companions throughout the perioperative period. In accordance with Article 2 of Resolution 736/2024^(1)^, it is essential that the PEP be grounded in a theoretical framework. This framework may be a nursing theory, a care framework, theoretical-conceptual structures, and others. However, given the complexity, diversity and specificity of surgeries and procedures in the perioperative period, a single theory may not fully support the PEP and it does not preclude integration of key protocols, such as safe surgery, patient safety goals, specific scales for predicting the risk of surgical positioning injury and anesthetic recovery and, evidently the psychosocial, psychobiological, and psychospiritual aspects involving the surgical patient (Figure 1).

The PEP can benefit from the Federal Nursing Council Resolution No. 696/2022, which regulates the role of Nursing in Digital Health and standardizes Telenursing^(10)^. This resolution expands the traditional practice of “preoperative assessment,” highlighting the rise of preoperative nursing teleconsultation, driven by restrictions imposed by the COVID-19 pandemic. Teleconsultation has proven to be an effective and safe alternative, comparable to in-person visits^(11)^. This opens a promising and relevant scenario for the work of perioperative nurses, with teleconsultation representing a possibility for carrying out this stage. Furthermore, nursing evaluations can also be carried out via telehealth, particularly for hospitalizations lasting less than 24 hours^(12)^.

Although the SAEP framework considers pre- or post-operative visits to be essential for quality perioperative nursing care, it is necessary to reflect that this practice in Brazil still represents a challenge for most Brazilian hospitals in terms of implementation. Several factors interfere with its adequate implementation, such as the inappropriate staffing in the OR, where this is not considered an activity belonging to the unit’s care practice; the high demand for concomitant clinical and administrative tasks; work overload; and lack of managerial support. All of these elements often prevent the OR nurse from traveling to perform pre- or post-operative visits.

However, it is worth highlighting that conducting the “patient assessment” through a preoperative visit by the OR nurse is a strategy considered the “gold standard” for perioperative NP. The preoperative visit reduces biases in clinical assessment, contributing to improving patient safety. When performed within 24 hours prior to the procedure, enables implementation of actions in a timely manner to improve patient’s readiness for the anesthetic-surgical process. The provision and preparation of materials for patients with allergies or special needs, collection of missing laboratory tests or blood typing and the waiting time for their results, and screening of resistant microorganisms for the adoption of infection prevention and control measures, when identified in advance, optimize patient care, operating room preparation, reduce surgical cancellation rates, and enhance perioperative care safety. Furthermore, the preoperative visit helps reduce patient anxiety and provides an opportunity to clarify doubts and involve the patient as an active participant of their care.

As the patient moves through different settings and is attended to by various professionals throughout the same experience, their assessment, the implementation of interventions and prescription of care, as well as the evaluation of their outcomes, occur at different times, or immediately after their execution/implementation. An example of this is the recommendation for special positioning devices for intraoperative use, a need that is identified preoperatively through a preoperative visit by the nurse, but whose effectiveness will be assessed in the immediate postoperative period, through evaluation of the patient in a postoperative visit to verify the absence or presence of pressure injuries resulting from surgical positioning.

A further challenge to the adequate execution of the PEP lies in the conduction of the postoperative visits or assessment, because a few Brazilian healthcare institutions have enough human resources for this activity in clinical practice, which is in turn delegated to the nurse at the inpatient unit admitting the patient postoperatively. Nevertheless, it would be important for the OR nurse to complete this stage, so that adverse events or inadequacies in the surgical care plan are not restricted to safety reports and quality indicators, allowing the nurse to actively participate in the evaluation of their practice to identify failures and improve the quality of perioperative care.

It should be noted that the nurse’s role is essential to integrate the PEP phases into the team when carrying out perioperative care^(4)^. The PEP aims to meet the needs of surgical patient^(5)^ by providing quality, individualized perioperative care, ensuring patient safety across all perioperative stages and settings. Furthermore, PEP encompasses nursing care management, including the management of environmental and personnel resources, with the provision of human and material resources necessary for a harm-free anesthetic-surgical procedure, and available according to the requirements of each anesthetic-surgical intervention^(4)^.

## CONCLUSION

This reflection recommends the adoption of the nomenclature “Perioperative Nursing Process” (PEP) to replace SAEP, enabling perioperative nurses to maintain the SAEP framework, while aligning with the updated COFEN Resolution on the Nursing Process. It should be noted that perioperative nurses play an essential role in the implementation of all phases of the PEP, by integrating the healthcare team in the delivering perioperative care across the immediate preoperative, transoperative/intraoperative and immediate postoperative/post-anesthetic recovery periods. Beyond a change in nomenclature, it is hoped that this article will encourage reflection on the challenges and opportunities of PEP in Brazil.

## DATA AVAILABILITY

Due to the methodological design, the dataset of this study is not publicly available.
