# Supplementary material for: Perioperative Nursing Process: reflection on updating the SAEP nomenclature
Source: Rev Esc Enferm USP. 2025 Oct 20;59:e20250165. doi: 10.1590/1980-220X-REEUSP-2025-0165en (PMC12543329; doi:10.1590/1980-220X-REEUSP-2025-0165en)
Supplement: Translation

# Translation: Processo de Enfermagem Perioperatória: reflexão sobre a atualização da nomenclatura SAEP

**DOI:** 10.1590/1980-220X-REEUSP-2025-0165pt

**Authors:** Juliana Rizzo Gnatta, Vanessa de Brito Poveda, Elaine Ribeiro, Erika Christiane Marocco Duran, Camila Takao Lopes, Cassiane de Santana Lemos, Marla Andréia Garcia de Avila, Suzimar de Fátima Benato Fusco, Taysa de Fátima Garcia, Rita Catalina Aquino Caregnato

**Affiliations:** 1 Universidade de São Paulo, Escola de Enfermagem, Departamento de Enfermagem Médico-Cirúrgica, São Paulo, SP, Brasil.; 2 Universidade Estadual de Campinas, Faculdade de Enfermagem, Campinas, SP, Brasil.; 3 Universidade Federal de São Paulo, Escola Paulista de Enfermagem, São Paulo, SP, Brasil.; 4 Universidade Estadual Paulista, Faculdade de Medicina de Botucatu, Departamento de Enfermagem, Botucatu, SP, Brasil.; 5 Universidade Federal de Minas Gerais, Belo Horizonte, MG, Brasil.; 6 Universidade Federal de Ciências da Saúde de Porto Alegre, Departamento de Enfermagem, Porto Alegre, RS, Brasil.

**Keywords:** Enfermagem, Enfermagem Perioperatória, Período Perioperatório, Processo de Enfermagem, Sistematização da Assistência de Enfermagem

## Abstract

**Objetivo:**

Refletir sobre a relevância da Sistematização da Assistência de Enfermagem na prática perioperatória (SAEP) do enfermeiro, comparando-a à Resolução nº 736 de 2024 do Conselho Federal de Enfermagem e propor a atualização de sua nomenclatura para garantir conformidade com a Resolução mencionada.

**Método:**

Trata-se de um estudo reflexivo em que especialistas na temática perioperatória e/ou de Processo de Enfermagem (PE) reuniram-se para discutir o modelo SAEP, adotado pela enfermagem perioperatória brasileira desde a década de 1990. São discutidos aspectos históricos sobre o modelo conceitual “Sistemática de Enfermagem Perioperatória”, abordando sua relevância na prática perioperatória do enfermeiro e comparando-o com a Resolução nº 736/2024 do Conselho Federal de Enfermagem (COFEN).

**Resultados:**

O modelo SAEP proposto na década de 90 ainda é atual na prática de enfermagem perioperatória, visto que nele são contempladas as etapas do PE em sua totalidade. Considerando sua importância, atualidade e aplicabilidade na prática do enfermeiro perioperatório, são estabelecidos paralelos com as etapas do PE descrito na Resolução nº 736, sugerindo uma nova nomenclatura: Processo de Enfermagem Perioperatória (PEP).

**Conclusão:**

Sugere-se a atualização da nomenclatura de SAEP para PEP, ao alinhar o modelo conceitual da SAEP, a Resolução COFEN 736/2024 e a terminologia “Processo de Enfermagem” adotada globalmente.

## INTRODUÇÃO

Em Janeiro de 2024, o Conselho Federal de Enfermagem (COFEN) brasileiro publicou a Resolução 736, que dispõe sobre a implementação do Processo de Enfermagem (PE) em todo contexto socioambiental onde ocorre o cuidado de enfermagem^(1)^>. Essa publicação resultou na revogação da Resolução COFEN 358/2009, que dispunha sobre a Sistematização da Assistência de Enfermagem (SAE) e a implementação do PE em ambientes, públicos ou privados, em que ocorre o cuidado profissional de Enfermagem, e dava outras providências^(2)^.

A Resolução revogada, 358/2009, definia a SAE como a organização do trabalho profissional quanto ao método, pessoal e instrumentos, tornando possível a operacionalização do PE. No entanto, todo o seu conteúdo referia-se ao PE, definindo-o como “um instrumento metodológico que orienta o cuidado profissional de Enfermagem e a documentação da prática profissional”^(2)^.

Pelo fato de a SAE ser um atributo gerencial/organizativo, ou seja, dependente da gestão em enfermagem, além de ser um conceito considerado imaturo^(3)^, o grupo de trabalho nomeado pelo COFEN para elaboração da Resolução 736/2024 decidiu por não abordar a SAE. A atual Resolução apresenta o PE como metodologia para conduzir o trabalho do enfermeiro, fundamentado no pensamento crítico, reflexivo e julgamento clínico, permitindo implementar na prática assistencial os conhecimentos técnico-científicos direcionados ao cuidado do indivíduo, da coletividade e de grupos, norteando a gestão do cuidado^(1)^.

Anteriormente à publicação da Resolução COFEN 358/2009^(2)^, Jouclas e Castellanos, em 1990, idealizaram o modelo denominado “Sistemática de Assistência de Enfermagem Perioperatória” (SAEP), com a proposta de uma avaliação holística do paciente, estabelecendo os cuidados de maneira individualizada e centrados nas reais necessidades do paciente cirúrgico^(4)^, sendo posteriormente denominado nacionalmente como “Sistematização da Assistência de Enfermagem Perioperatória”^(5)^.

A Enfermagem Perioperatória brasileira está familiarizada com o termo SAEP, implementando tal modelo para prestar assistência integral ao paciente cirúrgico em todas as fases do período perioperatório (pré-operatório, intraoperatório e pós-operatório) de forma continuada, participativa, individualizada, documentada e avaliada^(4)^. Entretanto, dada a confusão conceitual entre SAE e PE ao longo das décadas, a partir da nova Resolução nº 736/2024, é relevante a reflexão sobre a atualização da nomenclatura “SAEP”. A confusão conceitual mencionada entre a SAE e o PE tem levado os profissionais a utilizarem os termos de forma intercambiável, dificultando seu entendimento e diferenciação^(6-8)^. Na prática clínica, esta confusão projeta o PE não como um guia intelectual ou a aplicação do método científico na prática de enfermagem, mas como um conjunto burocrático de tarefas ou documentação para o qual frequentemente faltam recursos adequados. Tal percepção pode desmotivar os enfermeiros assistenciais a apropriarem-se do uso adequado do método do PE e desencorajar os gestores a buscarem recursos suficientes para sua implementação.

Ressalta-se que a expressão SAE existe somente no Brasil, visto que, internacionalmente, ao se referir ao PE, utiliza-se “*Nursing Process”*, expressão também indexada como descritor nas bases de dados científicas. “O conceito de Sistematização da Assistência de Enfermagem (SAE) surgiu no Brasil, com a premissa de que seria o contexto no qual a operacionalização do PE aconteceria”^(9)^. Nesse sentido, a atual Resolução do COFEN, ao eliminar do seu texto o termo SAE e direcionar-se ao PE, provocou o seguinte questionamento nos enfermeiros com *expertise* perioperatória: qual seria a nomenclatura indicada para representar os elementos veiculados pela SAEP, utilizada pela enfermagem perioperatória brasileira? Nesse cenário, especialistas reuniram-se para discutir essa questão e definiram como objetivo refletir sobre a relevância da SAEP, comparando-a à Resolução nº 736 de 2024 do COFEN, e propor a atualização de sua nomenclatura para garantir conformidade com a Resolução mencionada.

## MÉTODO

### Desenho de Estudo

Trata-se de estudo reflexivo construído por um grupo de enfermeiros com expertise na temática perioperatória e/ou de Processo de Enfermagem. Alguns são membros da Diretoria da Associação Brasileira de Enfermeiros de Centro Cirúrgico, Recuperação Anestésica e Centro de Material e Esterilização (SOBECC), da gestão 2023-2024 e outros, membros da Rede de Pesquisa em Processo de Enfermagem (RePPE) e da Comissão de Sistematização da Prática de Enfermagem (COMSISTE) da Associação Brasileira de Enfermagem (ABEn). Discutem-se aspectos históricos sobre o modelo conceitual proposto por Castellanos e Jouclas^(4)^, adotado pela enfermagem perioperatória brasileira, conhecido como SAEP, abordando sua relevância na prática perioperatória do enfermeiro e comparando-o com a Resolução nº 736 do COFEN, publicada em 2024^(1)^. Finalmente, propõe-se a adoção de uma nomenclatura para atender à Resolução atual, em substituição à SAEP.

### Aspectos Éticos

Por tratar-se de um artigo de reflexão, sem envolver seres humanos, este manuscrito dispensa a necessidade de aprovação em Comitê de Ética e Pesquisa e de Termo de Consentimento Livre e Esclarecido.

## RESULTADOS

Profissionais de enfermagem brasileiros, ao utilizar a terminologia SAEP, consideravam que essa nomenclatura expressava o PE, englobando todo o período perioperatório e destacando a interconexão intrínseca entre as três fases: pré-operatória, intraoperatória e pós-operatória. No contexto da SAEP, o PE seria usado como uma ferramenta para instrumentalizar o enfermeiro a prestar o atendimento com continuidade, levantando as necessidades individuais, identificando as respostas humanas e definindo os cuidados específicos em cada etapa do período perioperatório.

O modelo da SAEP recomenda que a “Avaliação do Pré-operatória” seja feita por meio da visita pré-operatória realizada pelo enfermeiro do Centro Cirúrgico, em algum momento durante as 24 horas que antecedem o procedimento anestésico-cirúrgico^(4)^. Dessa forma, a partir do momento em que a visita pré-operatória começa a ser realizada, inicia-se, de fato, o Processo de Enfermagem Perioperatória. Esta etapa é equivalente à primeira etapa do PE proposta pela Resolução 736/2024, denominada “avaliação de enfermagem”^(1)^, e tem o objetivo de realizar a anamnese, o exame físico e a observação direta, avaliar os resultados de exames laboratoriais e de imagem, identificar as necessidades e especificidades de assistência durante o transoperatório.

Assim, a etapa de “avaliação pré-operatória” começa com o enfermeiro do Centro Cirúrgico indo presencialmente até a unidade de internação onde está o paciente, ou conectando-se a ele por meio de alguma modalidade de tecnologia de informação e comunicação, como a telenfermagem. O enfermeiro perioperatório realiza a avaliação por meio da anamnese, do exame físico e da avaliação de exames, e registra-os. Inicia-se a segunda etapa, que conforme consta na Resolução 736/2024 é denominada “Diagnóstico de Enfermagem”. A partir da identificação dos problemas, vulnerabilidades ou disposições para melhorar comportamentos de saúde do paciente, são elencados os diagnósticos de enfermagem que subsidiam a elaboração do “Planejamento de cuidados” individualizado a ser implementado no pré e transoperatório, atendendo e respeitando as especificidades do paciente que será submetido ao procedimento anestésico-cirúrgico. Este momento corresponde à terceira etapa do PE, denominada “Planejamento de Enfermagem”.

Ao receber o paciente que já foi avaliado por meio da visita pré-operatória presencial ou por telenfermagem, o enfermeiro que realiza a admissão no CC recebe informações e analisa todas as etapas do PE já realizadas e documentadas. Além disso, na recepção do paciente, o enfermeiro avalia e registra as condições do paciente no dia da cirurgia, decide e documenta a manutenção e/ou modificação de diagnósticos de enfermagem identificados, e inicia a “Implementação da Assistência”, o que corresponde à quarta etapa do PE – a “Implementação de Enfermagem”.

Ao finalizar o período intraoperatório, o enfermeiro do CC documenta a “Evolução de Enfermagem” (quinta etapa do PE) e o paciente é encaminhado para a Sala de Recuperação Pós-Anestésica (SRPA). Na SRPA, o paciente é recebido pelo enfermeiro do setor, o qual recebe informações da equipe interprofissional e analisa todos os registros das etapas do PE realizadas desde a avaliação pré-operatória do paciente até o término do transoperatório. O enfermeiro aplica todas as etapas do PE. Ele avalia e registra novamente as condições clínicas do paciente, eventuais novos problemas e vulnerabilidades identificadas, definindo ou redefinindo as condutas que devem ser adotadas no pós-operatório imediato, por meio da prescrição de enfermagem. Na alta da SRPA, o enfermeiro realiza a evolução de enfermagem e ocorre a transferência de cuidado para a unidade de destino, onde o profissional de enfermagem da respectiva unidade continuará os cuidados, guiados pelo PE. Cabe destacar que pacientes submetidos a cirurgias que requerem cuidados críticos no pós-operatório imediato são transferidos diretamente do CC para a Unidade de Terapia Intensiva, respeitando a continuidade das mesmas etapas do PE.

Destaca-se que no modelo SAEP de Castellanos e Jouclas^(4)^, é recomendado que o enfermeiro do CC realize a visita pós-operatória, permitindo a reavaliação de todos os cuidados propostos e análise dos resultados obtidos em todo o período perioperatório. O nível de satisfação do paciente e possíveis intercorrências identificadas devem ser registradas, como, por exemplo, a evidência de uma lesão por posicionamento cirúrgico inadequado.

Nesta perspectiva, é importante esclarecer que o modelo SAEP proposto na década de 90 ainda é atual na prática de enfermagem perioperatória, visto que nele são contempladas as etapas do PE em sua totalidade, bem como a necessidade de estar amparado em um suporte/referencial teórico respeitando os metaparadigmas da profissão: ambiente, saúde, enfermagem e pessoa. Adicionalmente, os aspectos gerenciais do cuidado perioperatório são relacionados neste sistema, conforme apresentado na Figura 1.

**Figura 1:** Mapeamento da evolução do Processo de Enfermagem Perioperatória segundo o Modelo SAEP^(4)^ e sua interlocução com o Processo de Enfermagem segundo a Resolução COFEN 736/2024^(1)^. São Paulo, 2025.

Na Figura 1, evidencia-se a magnitude e a complexidade do modelo SAEP e sua correspondência com as etapas do PE propostas pela Resolução COFEN 736/2024.

A execução completa do PE aplicado às necessidades do paciente mapeia as necessidades de maneira integral e individualizada. Isso permite a organização dos recursos humanos e materiais em conformidade com as especificidades da cirurgia, demandas do paciente e da equipe cirúrgica, além de amparar o enfermeiro em todo o planejamento perioperatório, desde a transferência de cuidado. Cabe destacar que o registro sistematizado das etapas do PE cria a possibilidade da monitorização de indicadores assistenciais, clínicos e financeiros, o que, sob esta perspectiva, revela também a relação entre o PE e a segurança do paciente cirúrgico.

Frente a essa reflexão, propõe-se a adoção da nomenclatura “Processo de Enfermagem Perioperatória (PEP)” em substituição à SAEP, visto que o PEP mantém consistência com o modelo conceitual da SAEP e organiza-se em cinco etapas interrelacionadas (Figura 2), as quais correspondem às etapas do PE, conforme apresentado na Figura 1.

**Figura 2:** Estrutura do Processo de Enfermagem Perioperatória (PEP). São Paulo, 2025.

Conforme apresentado na Figura 2, o PEP deve ser aplicado a cada uma das três fases que compõem o período perioperatório (pré, trans e pós-operatório), de forma continuada e avaliada, garantindo uma assistência integral, participativa, continuada e individualizada ao paciente cirúrgico.

## DISCUSSÃO

A Resolução nº 736^(1)^ determina que o PE deve ser implementado em todo contexto em que ocorre o cuidado de enfermagem, portanto, inclui a assistência ao paciente cirúrgico. A Resolução reforça as cinco etapas do PE inter-relacionadas, interdependentes, recorrentes e cíclicas, sendo elas: 1. “Avaliação de Enfermagem”, momento caracterizado pela coleta de dados subjetivos (entrevista) e objetivos (exame físico, exames laboratoriais e de imagem) para a obtenção de informações pertinentes sobre as necessidades do cuidado de Enfermagem; 2. “Diagnóstico de Enfermagem”, que estabelece, por meio do julgamento clínico das informações obtidas na etapa anterior, os problemas existentes e condições de vulnerabilidades ou disposições para melhorar comportamentos de saúde; 3. “Planejamento de Enfermagem”, no qual se desenvolve plano assistencial direcionado para a pessoa, família, coletividade ou grupos especiais, compartilhado com os sujeitos do cuidado, equipe de Enfermagem e saúde; 4. “Implementação de Enfermagem”, contempla a execução, pela equipe de enfermagem, das intervenções, ações e atividades previstas por meio do planejamento assistencial; 5. “Evolução de Enfermagem”, verifica os resultados de enfermagem alcançados e identifica o estado de saúde da pessoa, família, coletividade e grupos especiais, permitindo ao enfermeiro analisar e revisar todo o PE, retornando à primeira etapa^(1)^. Na prática assistencial, observa-se que o Modelo SAEP aplica essas cinco etapas de forma continuada e avaliada durante as três etapas do perioperatório (Figura 2).

A SAEP, proposta por Castellanos e Jouclas^(4)^, possui interlocução com a Resolução 736/2024^(1)^, uma vez que a “avaliação pré-operatória” inclui a coleta de dados realizada na “avaliação de enfermagem” e, que no caso do período pré-operatório, contempla a visita pré-operatória de enfermagem; a “identificação de problemas” corresponde à definição dos “diagnósticos de enfermagem”; o “planejamento de cuidados” é desenvolvido por meio do “planejamento de enfermagem”; a “implementação da assistência de enfermagem para o período transoperatório” refere-se à “implementação de enfermagem” propriamente dita; e, por fim, a “avaliação da assistência pós-operatória” equivale à “evolução de enfermagem”, fase destinada à avaliação da assistência planejada e implementada, incluindo a realização da “visita pós-operatória de enfermagem” no período pós-operatório imediato (Figura 1).

O PEP pode ser definido como o eixo estruturante do cuidado da Enfermagem Perioperatória, um método que orienta o pensamento crítico e o julgamento clínico do Enfermeiro das unidades de internação pré e pós-operatórias e CC, direcionando a equipe de enfermagem para o cuidado à pessoa, família e acompanhante que vivenciam o período perioperatório. É fundamental que o PEP seja norteado por um suporte teórico, em consonância com o Artigo 2º da Resolução 736/2024^(1)^. Tal referencial pode ser uma teoria de enfermagem, um modelo de cuidado, estruturas teórico-conceituais, entre outros. Contudo, é pertinente compreender que, dada a complexidade, diversidade e especificidade das cirurgias e procedimentos que ocorrem no perioperatório, somente uma teoria pode não ser capaz de nortear o PEP e seu uso não exclui a integração de protocolos fundamentais, como o de cirurgia segura, as metas de segurança do paciente, as escalas específicas de predição de risco de lesão de posicionamento cirúrgico e de recuperação anestésica e, evidentemente, os aspectos psicossociais, psicobiológicos e psicoespirituais que envolvem o paciente cirúrgico (Figura 1).

O PEP pode ser beneficiado pela Resolução do Conselho Federal de Enfermagem Nº 696/2022, que regulamenta a atuação da Enfermagem na Saúde Digital e normatiza a Telenfermagem^(10)^. Essa resolução complementa a prática tradicional da “avaliação pré-operatória”, destacando a ascensão da teleconsulta de enfermagem no pré-operatório, impulsionada pelas restrições impostas pela pandemia de COVID-19. A teleconsulta tem se mostrado uma alternativa eficaz e segura, comparável à visita presencial^(11)^. Dessa maneira, observa-se um cenário promissor e relevante para atuação do enfermeiro perioperatório, com a teleconsulta representando uma possibilidade para a execução dessa etapa. Outrossim, a evolução de enfermagem também pode ser realizada por meio de telessaúde, principalmente nos casos com internações inferiores a 24 horas^(12)^.

Embora o Modelo SAEP preconize a realização das visitas, pré ou pós-operatórias, como partes integrantes essenciais de uma assistência de enfermagem perioperatória com qualidade, é necessário refletir que esta prática no Brasil ainda representa um desafio para a maioria dos hospitais brasileiros em termos de execução. Diversos fatores interferem na sua adequada realização, como o dimensionamento inapropriado de recursos humanos no CC, que não prevê esta atividade constituindo a prática assistencial da unidade, a alta demanda de funções assistenciais e administrativas concomitantes, a sobrecarga de trabalho, a falta de apoio de lideranças. Todos estes elementos frequentemente impedem que o enfermeiro do CC se desloque para realizar as visitas pré ou pós-operatórias.

Entretanto, destaca-se que a realização da “avaliação do paciente” por meio da visita pré-operatória realizada pelo enfermeiro do Centro Cirúrgico é estratégia considerada “padrão ouro” para o PE perioperatório. A visita pré-operatória reduz vieses na avaliação clínica, contribuindo para melhorar a segurança do paciente e, quando realizada até 24 horas antes do procedimento, permite que ações sejam implementadas em tempo hábil no sentido de melhorar as condições do paciente para o processo anestésico-cirúrgico. Provisão e previsão de materiais para pacientes alérgicos ou com necessidades especiais, coleta de exames laboratoriais faltantes ou tipagem sanguínea e tempo de aguardo para seus resultados, triagem de microrganismos resistentes para a adoção de medidas de prevenção e controle de infecções, identificados com antecedência, otimizam a assistência ao paciente, o preparo da sala operatória, reduzem as taxas de suspensões cirúrgicas e aumentam a segurança da assistência perioperatória. Além disso, a visita pré-operatória contribui para reduzir a ansiedade do paciente e propicia um momento para o esclarecimento de dúvidas e envolvimento do paciente como parte integrante de seu cuidado.

Como o paciente, ao longo da mesma experiência, transita em diferentes espaços e é atendido por diversos profissionais, sua avaliação, a implementação de intervenções e prescrição do cuidado, bem como a avaliação de seus resultados, acontecem em tempos distintos, ou imediatamente após sua realização/implementação. Exemplifica este aspecto a recomendação de posicionadores especiais para o intraoperatório, necessidade que é identificada no pré-operatório por meio da visita pré-operatória realizada pelo enfermeiro, mas que terá sua efetividade avaliada no pós-operatório imediato, mediante avaliação do paciente por meio da visita pós-operatória para verificar ausência ou presença de lesões por pressão por posicionamento cirúrgico.

Outra fragilidade observada na adequada execução do PEP também pode ser observada na realização da visita ou avaliação pós-operatória, pois que na prática clínica identifica-se que poucas instituições de saúde brasileiras disponibilizam recursos humanos para esta atividade, que fica restrita ao enfermeiro da unidade de internação que recebe o paciente no período pós-operatório. Contudo, seria importante o enfermeiro do CC concluir esta etapa, para que eventos adversos ou inadequações do plano assistencial cirúrgico não ficassem restritos apenas a notificações de relatórios de segurança e de indicadores de qualidade, permitindo ao enfermeiro participar de forma ativa da avaliação de sua práxis a fim de identificar as falhas e melhorar a qualidade assistencial perioperatória.

Destaca-se que a atuação do enfermeiro é imprescindível para integrar as fases do PEP à equipe na realização dos cuidados perioperatórios^(4)^. O PEP objetiva a satisfação das necessidades da pessoa em condição cirúrgica^(5)^, oferecendo um cuidado perioperatório de qualidade, individualizado, garantindo a segurança do paciente em todas as etapas e cenários perioperatórios. Além disso, o PEP envolve a gestão da assistência de enfermagem, e contempla o gerenciamento de recursos ambientais e de pessoal, com a provisão de recursos humanos e materiais necessários ao ato anestésico-cirúrgico, para o uso livre de danos e disponibilidade de acordo com as necessidades de cada intervenção anestésico-cirúrgica^(4)^.

## CONCLUSÃO

A presente reflexão recomenda a adoção da nomenclatura “Processo de Enfermagem Perioperatória” (PEP) em substituição à SAEP, ao permitir à enfermagem perioperatória a manutenção do modelo proposto na SAEP, ao mesmo tempo em que estará em consonância com a Resolução atualizada do COFEN, que dispõe sobre o Processo de Enfermagem. Destaca-se que o enfermeiro perioperatório é fundamental para a aplicação de todas as fases do PEP, ao integrar a equipe de saúde na realização dos cuidados perioperatórios, que abrangem os períodos pré-operatório imediato, transoperatório/intraoperatório e pós-operatório imediato/recuperação pós-anestésica. Espera-se que, para além da alteração da nomenclatura, este artigo possa promover uma reflexão acerca dos desafios e potencialidades do PEP no Brasil.

## DISPONIBILIDADE DE DADOS

Devido ao desenho metodológico, o conjunto de dados que dá suporte aos resultados deste estudo não está disponível publicamente.

# Translation: Proceso de enfermería perioperatoria: reflexión sobre la actualización de la nomenclatura SAEP

**DOI:** 10.1590/1980-220X-REEUSP-2025-0165es

**Authors:** Juliana Rizzo Gnatta, Vanessa de Brito Poveda, Elaine Ribeiro, Erika Christiane Marocco Duran, Camila Takao Lopes, Cassiane de Santana Lemos, Marla Andréia Garcia de Avila, Suzimar de Fátima Benato Fusco, Taysa de Fátima Garcia, Rita Catalina Aquino Caregnato

**Affiliations:** 1 Universidade de São Paulo, Escola de Enfermagem, Departamento de Enfermagem Médico-Cirúrgica, São Paulo, SP, Brasil.; 2 Universidade Estadual de Campinas, Faculdade de Enfermagem, Campinas, SP, Brasil.; 3 Universidade Federal de São Paulo, Escola Paulista de Enfermagem, São Paulo, SP, Brasil.; 4 Universidade Estadual Paulista, Faculdade de Medicina de Botucatu, Departamento de Enfermagem, Botucatu, SP, Brasil.; 5 Universidade Federal de Minas Gerais, Belo Horizonte, MG, Brasil.; 6 Universidade Federal de Ciências da Saúde de Porto Alegre, Departamento de Enfermagem, Porto Alegre, RS, Brasil.

**Keywords:** Enfermería, Enfermería Perioperatoria, Periodo Perioperatorio, Proceso de Enfermería, Atención de Enfermería

## Abstract

**Objetivo:**

Reflexionar sobre la relevancia de la Sistematización de la Atención de Enfermería brasileña en la práctica perioperatoria (SAEP) de enfermería, comparándola con la Resolución No. 736 de 2024 del Consejo Federal de Enfermería (COFEN) y proponiendo la actualización de su nomenclatura para asegurar el cumplimiento de la citada Resolución.

**Método:**

Se trata de un estudio reflexivo en el que especialistas en temas perioperatorios y/o Proceso de Enfermería (PE) se reunieron para discutir el modelo denominado SAEP, adoptado por la enfermería perioperatoria brasileña desde la década de 1990. Se discuten aspectos históricos del modelo conceptual “Sistemática de Enfermería Perioperatoria”, abordando su relevancia en la práctica perioperatoria de los enfermeros y comparándolo con la Resolución nº 736/2024 del Consejo Federal de Enfermería.

**Resultados:**

El modelo SAEP propuesto en la década de 1990 sigue vigente en la práctica de enfermería perioperatoria, ya que abarca las etapas del PE en su totalidad. Considerando su importancia, estado actual y aplicabilidad en la práctica de la enfermería perioperatoria, se establecen paralelismos con las etapas del PE descritas en la Resolución Nº 736, sugiriendo una nueva nomenclatura: Proceso de enfermería perioperatoria (PEP).

**Conclusión:**

Se sugiere actualizar la nomenclatura de SAEP a PEP, alineando el modelo conceptual de la SAEP, la Resolución COFEN nº 736 de 2024 y la terminología “Proceso de Enfermería” adoptada globalmente.

## INTRODUCCIÓN

En enero de 2024, el Consejo Federal de Enfermería (COFEN) de Brasil publicó la Resolución 736, que dispone sobre la implementación del Proceso de Enfermería (PE) en todos los contextos socioambientales donde se presta atención de enfermería^(1)^. Esta publicación dio lugar a la derogación de la Resolución COFEN 358/2009, que regulaba la Sistematización de la Asistencia de Enfermería (SAE) y la implementación del PE en entornos públicos o privados en los que se prestan cuidados de enfermería profesionales, y establecía otras disposiciones^(2)^.

La resolución derogada, 358/2009, definía la SAE como la organización del trabajo profesional en cuanto al método, el personal y los instrumentos, lo que hacía posible la puesta en práctica del PE. Sin embargo, todo su contenido se refería al PE, definiéndolo como “un instrumento metodológico que orienta la atención profesional de enfermería y la documentación de la práctica profesional”^(2)^.

Debido a que la SAE es un atributo gerencial/organizativo, es decir, dependiente de la gestión en enfermería, además de ser un concepto considerado inmaduro^(3)^, el grupo de trabajo designado por el COFEN para elaborar la Resolución 736/2024 decidió no abordar la SAE. La Resolución actual presenta el PE como metodología para dirigir el trabajo del enfermero, basado en el pensamiento crítico, reflexivo y el juicio clínico, permitiendo implementar en la práctica asistencial los conocimientos técnico-científicos dirigidos al cuidado del individuo, de la colectividad y de los grupos, orientando la gestión del cuidado^(1)^.

Antes de la publicación de la Resolución COFEN 358/2009^(2)^, Jouclas y Castellanos, en 1990, idealizaron el modelo denominado “Sistemática de Asistencia de Enfermería Perioperatoria” (SAEP), con la propuesta de una evaluación holística del paciente, estableciendo cuidados individualizados y centrados en las necesidades reales del paciente quirúrgico^(4)^, que posteriormente se denominó a nivel nacional “Sistematización de la Asistencia de Enfermería Perioperatoria”^(5)^.

La enfermería perioperatoria brasileña está familiarizada con el término SAEP, implementando dicho modelo para prestar asistencia integral al paciente quirúrgico en todas las fases del periodo perioperatorio (preoperatorio, intraoperatorio y postoperatorio) de forma continua, participativa, individualizada, documentada y evaluada^(4)^. Sin embargo, dada la confusión conceptual entre SAE y PE a lo largo de las décadas, a partir de la nueva Resolución n.º 736/2024, es relevante reflexionar sobre la actualización de la nomenclatura “SAEP”. La confusión conceptual mencionada entre SAE y PE ha llevado a los profesionales a utilizar los términos de forma intercambiable, lo que dificulta su comprensión y diferenciación^(6-8)^. En la práctica clínica, esta confusión proyecta el PE no como una guía intelectual o la aplicación del método científico en la práctica de la enfermería, sino como un conjunto burocrático de tareas o documentación para el que a menudo faltan recursos adecuados. Esta percepción puede desmotivar a los enfermeros asistenciales a apropiarse del uso adecuado del método del PE y desalentar a los gestores a buscar recursos suficientes para su implementación.

Cabe destacar que la expresión SAE solo existe en Brasil, ya que, a nivel internacional, cuando se hace referencia al PE, se utiliza “*Nursing Process”*, expresión también indexada como descriptor en las bases de datos científicas. “El concepto de Sistematización de la Asistencia de Enfermería (SAE) surgió en Brasil, con la premisa de que sería el contexto en el que se llevaría a cabo la operacionalización del PE”^(9)^. En este sentido, la actual Resolución del COFEN, al eliminar de su texto el término SAE y dirigirse al PE, provocó la siguiente pregunta entre los enfermeros con experiencia en el ámbito perioperatorio: ¿cuál sería la nomenclatura adecuada para representar los elementos transmitidos por la SAEP, utilizada por la enfermería perioperatoria brasileña? En este contexto, se reunieron especialistas para debatir esta cuestión y definieron como objetivo reflexionar sobre la relevancia de la SAEP, comparándola con la Resolución n.º 736 de 2024 del COFEN, y proponer la actualización de su nomenclatura para garantizar la conformidad con la Resolución mencionada.

## MÉTODO

### Diseño del Estudio

Se trata de un estudio reflexivo elaborado por un grupo de enfermeros con experiencia en el ámbito perioperatorio y/o en el proceso de enfermería. Algunos son miembros de la Junta Directiva de la Sociedad Brasileña de Enfermeros de Quirófano, Recuperación Anestésica y Centro de Material y Esterilización (SOBECC), de la gestión 2023-2024, y otros son miembros de la Red de Investigación en Procesos de Enfermería (RePPE) y de la Comisión de Sistematización de la Práctica de Enfermería (COMSISTE) de la Asociación Brasileña de Enfermería (ABEn). Se discuten aspectos históricos sobre el modelo conceptual propuesto por Castellanos y Jouclas^(4)^, adoptado por la enfermería perioperatoria brasileña, conocido como SAEP, abordando su relevancia en la práctica perioperatoria del enfermero y comparándolo con la Resolución n.º 736 del COFEN, publicada en 2024^(1)^. Finalmente, se propone la adopción de una nomenclatura para cumplir con la Resolución actual, en sustitución de la SAEP.

### Aspectos Éticos

Al tratarse de un artículo de reflexión, sin involucrar a seres humanos, este manuscrito no requiere la aprobación de un Comité de Ética y Investigación ni un Término de Consentimiento Libre y Esclarecido.

## RESULTADOS

Los profesionales de enfermería brasileños, al utilizar la terminología SAEP, consideraban que esta nomenclatura expresaba el PE, abarcando todo el período perioperatorio y destacando la interconexión intrínseca entre las tres fases: preoperatoria, intraoperatoria y postoperatoria. En el contexto de la SAEP, el PE se utilizaría como una herramienta para capacitar al enfermero para prestar una atención continua, determinando las necesidades individuales, identificando las respuestas humanas y definiendo los cuidados específicos en cada etapa del período perioperatorio.

El modelo SAEP recomienda que la “Evaluación Preoperatoria” se realice mediante una visita preoperatoria realizada por el enfermero del Centro de Cirugía (CC), en algún momento durante las 24 horas previas al procedimiento anestésico-quirúrgico^(4)^. De este modo, desde el momento en que comienza la visita preoperatoria, se inicia, de hecho, el Proceso de Enfermería Perioperatoria. Esta etapa es equivalente a la primera etapa del PE propuesta por la Resolución 736/2024, denominada “evaluación de enfermería”^(1)^, y tiene como objetivo realizar la anamnesis, el examen físico y la observación directa, evaluar los resultados de los exámenes de laboratorio y de imagen, identificar las necesidades y especificidades de la asistencia durante el perioperatorio.

Así, la etapa de “evaluación preoperatoria” comienza con la visita del enfermero del CC a la unidad de hospitalización donde se encuentra el paciente, o conectándose con él a través de algún medio de tecnología de la información y la comunicación, como la tele-enfermería. El enfermero perioperatorio realiza la evaluación mediante la anamnesis, el examen físico y la evaluación de los exámenes, y los registra. Se inicia la segunda etapa, que según la Resolución 736/2024 se denomina “Diagnóstico de Enfermería”. A partir de la identificación de los problemas, vulnerabilidades o disposiciones para mejorar los comportamientos de salud del paciente, se enumeran los diagnósticos de enfermería que sustentan la elaboración del “Plan de cuidados” individualizado que se implementará en el pre y transoperatorio, atendiendo y respetando las especificidades del paciente que se someterá al procedimiento anestésico-quirúrgico. Este momento corresponde a la tercera etapa del PE, denominada “Planificación de enfermería”.

Al recibir al paciente que ya ha sido evaluado mediante la visita preoperatoria presencial o por tele-enfermería, el enfermero que realiza el ingreso en el CC recibe información y analiza todas las etapas del PE ya realizadas y documentadas. Además, al recibir al paciente, el enfermero evalúa y registra su estado el día de la cirugía, decide y documenta el mantenimiento y/o la modificación de los diagnósticos de enfermería identificados e inicia la “Implementación de la Asistencia”, que corresponde a la cuarta etapa del PE: la “Implementación de Enfermería”.

Al finalizar el periodo intraoperatorio, el enfermero del CC documenta la “Evolución de Enfermería” (quinta etapa del PE) y el paciente es trasladado a la Sala de Recuperación Postanestésica (SRPA). En la SRPA, el paciente es recibido por el enfermero del sector, quien recibe información del equipo interprofesional y analiza todos los registros de las etapas del PE realizadas desde la evaluación preoperatoria del paciente hasta el final del transoperatorio. El enfermero aplica todas las etapas del PE. Evalúa y registra nuevamente las condiciones clínicas del paciente, los posibles nuevos problemas y vulnerabilidades identificados, definiendo o redefiniendo las conductas que deben adoptarse en el postoperatorio inmediato, mediante la prescripción de enfermería. Al alta de la SRPA, el enfermero realiza la evolución de enfermería y se produce el traslado de la atención a la unidad de destino, donde el profesional de enfermería de la unidad correspondiente continuará los cuidados, guiado por el PE. Cabe destacar que los pacientes sometidos a cirugías que requieren cuidados críticos en el postoperatorio inmediato son transferidos directamente del CC a la Unidad de Terapia Intensiva, respetando la continuidad de las mismas etapas del PE.

Cabe destacar que en el modelo SAEP de Castellanos y Jouclas^(4)^, se recomienda que el enfermero del CC realice la visita postoperatoria, lo que permite la reevaluación de todos los cuidados propuestos y el análisis de los resultados obtenidos durante todo el periodo perioperatorio. Se debe registrar el nivel de satisfacción del paciente y las posibles complicaciones identificadas, como, por ejemplo, la evidencia de una lesión por posicionamiento quirúrgico inadecuado.

En esta perspectiva, es importante aclarar que el modelo SAEP propuesto en la década de los 90 sigue siendo actual en la práctica de la enfermería perioperatoria, ya que contempla todas las etapas del PE, así como la necesidad de estar respaldado por un soporte/referencial teórico que respete los metaparadigmas de la profesión: entorno, salud, enfermería y persona. Además, los aspectos administrativos de la atención perioperatoria se relacionan en este sistema, tal y como se muestra en la Figura 1.

**Figura 1:** Mapeo de la evolución del Proceso de Enfermería Perioperatoria según el Modelo SAEP^(4)^ y su interlocución con el Proceso de Enfermería según la Resolución COFEN 736/2024^(1)^. São Paulo, 2025.

En la Figura 1 se evidencia la magnitud y la complejidad del modelo SAEP y su correspondencia con las etapas del PE propuestas por la Resolución COFEN 736/2024.

La ejecución completa del PE aplicado a las necesidades del paciente mapea las necesidades de manera integral e individualizada. Esto permite organizar los recursos humanos y materiales de acuerdo con las especificidades de la cirugía, las demandas del paciente y del equipo quirúrgico, además de apoyar al enfermero en toda la planificación perioperatoria, desde el traslado del paciente. Cabe destacar que el registro sistematizado de las etapas del PE crea la posibilidad de monitorear los indicadores asistenciales, clínicos y financieros, lo que, desde esta perspectiva, también revela la relación entre el PE y la seguridad del paciente quirúrgico.

Ante esta reflexión, se propone la adopción de la nomenclatura “Proceso de Enfermería Perioperatoria (PEP)” en sustitución de SAEP, ya que el PEP mantiene la coherencia con el modelo conceptual de la SAEP y se organiza en cinco etapas interrelacionadas (Figura 2), que corresponden a las etapas del PE, tal y como se presenta en la Figura 1.

**Figura 2:** Estructura del Proceso de Enfermería Perioperatoria (PEP). São Paulo, 2025.

Tal y como se muestra en la Figura 2, el PEP debe aplicarse a cada una de las tres fases que componen el periodo perioperatorio (preoperatorio, transoperatorio y postoperatorio), de forma continua y evaluada, garantizando una asistencia integral, participativa, continuada e individualizada al paciente quirúrgico.

## DISCUSIÓN

La Resolución n.º 736^(1)^ determina que el PE debe implementarse en todos los contextos en los que se presta atención de enfermería, por lo que incluye la asistencia al paciente quirúrgico. La Resolución refuerza las cinco etapas del PE, interrelacionadas, interdependientes, recurrentes y cíclicas, que son: 1. “Evaluación de Enfermería”, momento caracterizado por la recolección de datos subjetivos (entrevista) y objetivos (examen físico, exámenes de laboratorio y de imagen) para obtener información pertinente sobre las necesidades de cuidados de enfermería; 2. “Diagnóstico de enfermería”, que establece, mediante el juicio clínico de la información obtenida en la etapa anterior, los problemas existentes y las condiciones de vulnerabilidad o disposición para mejorar los comportamientos de salud; 3. “Planificación de enfermería”, en la que se desarrolla un plan de asistencia dirigido a la persona, la familia, la colectividad o grupos especiales, compartido con los sujetos de los cuidados, el equipo de enfermería y de salud; 4. “Implementación de enfermería”, contempla la ejecución, por parte del equipo de enfermería, de las intervenciones, acciones y actividades previstas mediante la planificación asistencial; 5. “Evolución de la enfermería”, verifica los resultados de enfermería alcanzados e identifica el estado de salud de la persona, la familia, la colectividad y los grupos especiales, lo que permite al enfermero analizar y revisar todo el PE, volviendo a la primera etapa^(1)^. En la práctica asistencial, se observa que el Modelo SAEP aplica estas cinco etapas de forma continua y evaluada durante las tres etapas del perioperatorio (Figura 2).

La SAEP, propuesta por Castellanos y Jouclas^(4)^, tiene interlocución con la Resolución 736/2024^(1)^, ya que la “evaluación preoperatoria” incluye la recolección de datos realizada en la “evaluación de enfermería” y, en el caso del período preoperatorio, contempla la visita preoperatoria de enfermería; la “identificación de problemas” corresponde a la definición de los “diagnósticos de enfermería”; la “planificación de los cuidados” se desarrolla mediante la “planificación de enfermería”; la “implementación de la asistencia de enfermería para el periodo transoperatorio” se refiere a la “implementación de enfermería” propiamente dicha; y, por último, la “evaluación de la asistencia posoperatoria” equivale a la “evolución de enfermería”, fase destinada a la evaluación de la asistencia planificada e implementada, incluida la realización de la “visita posoperatoria de enfermería” en el periodo posoperatorio inmediato (Figura 1).

El PEP puede definirse como el eje estructurante de la atención de enfermería perioperatoria, un método que orienta el pensamiento crítico y el juicio clínico del enfermero de las unidades de hospitalización pre y posoperatoria y de cuidados intensivos, dirigiendo al equipo de enfermería hacia la atención a la persona, la familia y el acompañante que viven el periodo perioperatorio. Es fundamental que el PEP se base en un soporte teórico, de conformidad con el artículo 2 de la Resolución 736/2024^(1)^. Este referente puede ser una teoría de enfermería, un modelo de atención, estructuras teórico-conceptuales, entre otros. Sin embargo, es pertinente comprender que, dada la complejidad, diversidad y especificidad de las cirugías y procedimientos que se realizan en el perioperatorio, una sola teoría puede no ser suficiente para orientar el PEP y su uso no excluye la integración de protocolos fundamentales, como el de cirugía segura, los objetivos de seguridad del paciente, las escalas específicas de predicción del riesgo de lesiones por posicionamiento quirúrgico y de recuperación anestésica y, evidentemente, los aspectos psicosociales, psicobiológicos y psicoespirituales que rodean al paciente quirúrgico (Figura 1).

El PEP puede beneficiarse de la Resolución del Consejo Federal de Enfermería N.º 696/2022, que regula la actuación de la enfermería en la salud digital y normaliza la tele-enfermería^(10)^. Esta resolución complementa la práctica tradicional de la “evaluación preoperatoria”, destacando el auge de la teleconsulta de enfermería en el preoperatorio, impulsada por las restricciones impuestas por la pandemia de COVID-19. La teleconsulta ha demostrado ser una alternativa eficaz y segura, comparable a la visita presencial^(11)^. De este modo, se observa un panorama prometedor y relevante para la actuación del enfermero perioperatorio, con la teleconsulta como una posibilidad para la ejecución de esta etapa. Además, la evolución de la enfermería también puede llevarse a cabo a través de la telesalud, especialmente en los casos con hospitalizaciones inferiores a 24 horas^(12)^.

Aunque el Modelo SAEP recomienda la realización de visitas pre y posoperatorias como parte integral de una asistencia de enfermería perioperatoria de calidad, es necesario reflexionar que esta práctica en Brasil todavía representa un reto para la mayoría de los hospitales brasileños en términos de ejecución. Diversos factores interfieren en su adecuada realización, como la dotación inadecuada de recursos humanos en el CC, que no prevé esta actividad como parte de la práctica asistencial de la unidad, la alta demanda de funciones asistenciales y administrativas concomitantes, la sobrecarga de trabajo y la falta de apoyo de los líderes. Todos estos elementos impiden con frecuencia que el enfermero del CC se desplace para realizar las visitas pre o posoperatorias.

Sin embargo, cabe destacar que la realización de la “evaluación del paciente” mediante la visita preoperatoria realizada por el enfermero del CC es una estrategia considerada “estándar de oro” para el PE perioperatorio. La visita preoperatoria reduce los sesgos en la evaluación clínica, lo que contribuye a mejorar la seguridad del paciente y, cuando se realiza hasta 24 horas antes del procedimiento, permite que se implementen medidas oportunas para mejorar las condiciones del paciente para el proceso anestésico-quirúrgico. El suministro y la previsión de materiales para pacientes alérgicos o con necesidades especiales, la recolección de exámenes de laboratorio pendientes o la tipificación sanguínea y el tiempo de espera de sus resultados, el cribado de microorganismos resistentes para la adopción de medidas de prevención y control de infecciones, identificados con antelación, optimizan la asistencia al paciente, la preparación del quirófano, reducen las tasas de suspensiones quirúrgicas y aumentan la seguridad de la asistencia perioperatoria. Además, la visita preoperatoria contribuye a reducir la ansiedad del paciente y proporciona un momento para aclarar dudas e involucrar al paciente como parte integrante de su cuidado.

Dado que el paciente, a lo largo de la misma experiencia, transita por diferentes espacios y es atendido por diversos profesionales, su evaluación, la implementación de intervenciones y la prescripción de cuidados, así como la evaluación de sus resultados, se producen en momentos distintos o inmediatamente después de su realización/implementación. Un ejemplo de este aspecto es la recomendación de posicionadores especiales para el intraoperatorio, una necesidad que se identifica en el preoperatorio mediante la visita preoperatoria realizada por el enfermero, pero cuya eficacia se evaluará en el postoperatorio inmediato, mediante la evaluación del paciente en la visita postoperatoria para verificar la ausencia o presencia de lesiones por presión debidas al posicionamiento quirúrgico.

Otra fragilidad observada en la ejecución adecuada del PEP también puede observarse en la realización de la visita o evaluación postoperatoria, ya que en la práctica clínica se identifica que pocas instituciones de salud brasileñas disponen de recursos humanos para esta actividad, que se limita al enfermero de la unidad de hospitalización que recibe al paciente en el postoperatorio. Sin embargo, sería importante que el enfermero del CC concluyera esta etapa, para que los eventos adversos o las inadecuaciones del plan de asistencia quirúrgica no se limitaran únicamente a notificaciones de informes de seguridad e indicadores de calidad, permitiendo al enfermero participar activamente en la evaluación de su praxis con el fin de identificar las fallas y mejorar la calidad de la asistencia perioperatoria.

Cabe destacar que la actuación del enfermero es imprescindible para integrar las fases del PEP al equipo en la realización de los cuidados perioperatorios^(4)^. El PEP tiene como objetivo satisfacer las necesidades de la persona en condiciones quirúrgicas^(5)^, ofreciendo una atención perioperatoria de calidad e individualizada, garantizando la seguridad del paciente en todas las etapas y escenarios perioperatorios. Además, el PEP implica la gestión de la asistencia de enfermería y contempla la gestión de los recursos ambientales y de personal, con la provisión de los recursos humanos y materiales necesarios para el acto anestésico-quirúrgico, para su uso sin daños y su disponibilidad de acuerdo con las necesidades de cada intervención anestésico-quirúrgica^(4)^.

## CONCLUSIÓN

La presente reflexión recomienda la adopción de la nomenclatura “Proceso de Enfermería Perioperatoria” (PEP) en sustitución de SAEP, ya que permite a la enfermería perioperatoria mantener el modelo propuesto en SAEP, al tiempo que se ajusta a la Resolución actualizada del COFEN, que dispone sobre el Proceso de Enfermería. Cabe destacar que el enfermero perioperatorio es fundamental para la aplicación de todas las fases del PEP, al integrar al equipo de salud en la realización de los cuidados perioperatorios, que abarcan los periodos preoperatorio inmediato, transoperatorio/intraoperatorio y postoperatorio inmediato/recuperación postanestésica. Se espera que, además del cambio de nomenclatura, este artículo promueva una reflexión sobre los retos y el potencial del PEP en Brasil.

## DISPONIBILIDAD DE DATOS

Debido al diseño metodológico, el conjunto de datos que respalda los resultados de esta investigación no está disponible publicamente.
